# Outcomes Following Institutional Adoption of Vacuum-Assisted Micro-ECIRS for Symptomatic 1–2 cm Lower Pole Renal Stones: A Retrospective Non-Concurrent Cohort Study

**DOI:** 10.3390/jcm15218534

**Published:** 2026-11-02

**Authors:** Hüseyin Biçer, Abdullah Gölbaşı, Burak Elmaağaç, Ali Yasin Özercan, Hasan Oruç Eroğlu, Mert Ali Karadağ

**Affiliations:** 1 Department of Urology, Kayseri City Hospital, 38080 Kayseri, Türkiye; 2 Department of Urology, Kayseri Faculty of Medicine, University of Health Sciences, 38080 Kayseri, Türkiye

**Keywords:** micro-percutaneous nephrolithotomy, micro-ECIRS, lower pole renal stones, suction ureteral access sheath, endoscopic combined intrarenal surgery, residual fragments, stone-free status

## Abstract

**Background/Objectives:** Micro-percutaneous nephrolithotomy (micro-PCNL) offers direct access to lower pole stones but has limited fragment evacuation. Vacuum-assisted micro-endoscopic combined intrarenal surgery (micro-ECIRS) adds flexible ureteroscopy and suction. We evaluated outcomes after institutional adoption of this technique for symptomatic 1–2 cm lower pole stones. **Methods:** This retrospective non-concurrent cohort included 81 consecutive adults treated between January 2016 and July 2025 (micro-ECIRS, *n* = 42; micro-PCNL, *n* = 39). Only micro-PCNL procedures (*n* = 33) preceded January 2021. One-month low-dose noncontrast computed tomography (earlier, pre-intervention, in four patients) classified outcomes as Grade A (no residual fragment), Grade B (none >2 mm), or Grade C (none >4 mm; main outcome). Comparisons were unadjusted. **Results:** Grade C was attained in 90.5% (micro-ECIRS) versus 64.1% (micro-PCNL) (absolute difference 26.4 percentage points, 95% confidence interval [CI] 8.2–43.1; risk ratio [RR] 1.41, 95% CI 1.09–1.82; *p* = 0.007) and Grade B in 71.4% versus 46.2% (*p* = 0.025). Grade A did not differ significantly (42.9% vs. 28.2%; *p* = 0.246). Secondary puncture (4.8% vs. 28.2%) and auxiliary procedures (4.8% vs. 25.6%) were less frequent after micro-ECIRS (both *p* < 0.05). Complications did not differ significantly (11.9% vs. 30.8%; *p* = 0.055). **Conclusions:** In this retrospective, predominantly non-concurrent cohort, institutional adoption of vacuum-assisted micro-ECIRS was associated with higher rates of no residual fragment larger than 2 mm or 4 mm and fewer additional punctures and subsequent procedures. A difference in complete stone clearance was not demonstrated. Treatment–era confounding, baseline imbalance, and complete-case selection prevent causal attribution to the technique.

## 1. Introduction

Lower pole renal stones remain a challenging subgroup in endourology because treatment success depends not only on effective stone fragmentation but also on the subsequent clearance of fragments from an anatomically dependent collecting system. A steep infundibulopelvic angle, narrow infundibular width, long infundibular length, and gravitational dependency may impair spontaneous fragment clearance after lithotripsy, regardless of the treatment modality used [1,2].

For symptomatic 1–2 cm lower pole stones, the optimal treatment strategy remains unsettled. Extracorporeal shock wave lithotripsy (ESWL), retrograde intrarenal surgery (RIRS), and percutaneous approaches are all used in clinical practice [1]. Comparative evidence suggests meaningful differences among these modalities in stone-free and retreatment outcomes, particularly for lower pole stones within this size range [2,3]. ESWL may be less effective in the presence of unfavorable lower pole anatomy or higher stone density, whereas RIRS may be technically limited by lower pole access, ureteroscope deflection, fragment migration, and incomplete fragment evacuation [1,2,4].

Micro-percutaneous nephrolithotomy (micro-PCNL), performed through a 4.85 Fr “all-seeing needle” without conventional tract dilation, was developed to provide direct percutaneous access while minimizing tract-related morbidity [5,6]. However, standard micro-PCNL has limited capacity for active fragment extraction, depends largely on passive drainage through a small-caliber ureteral catheter, and may provide limited access to fragments that migrate away from the initial percutaneous trajectory [6,7,8]. These limitations may be particularly relevant in the lower pole, where postoperative fragment clearance is strongly influenced by collecting-system anatomy. At the same time, the 4.85 Fr tract is less invasive than mini-PCNL: pooled analyses have shown comparable stone-free rates with a smaller hemoglobin decrease after micro-PCNL [9]. This favorable balance between access and invasiveness was the reason micro-PCNL was the preferred percutaneous option for these stones in our institution and the basis on which the combined technique was developed.

Endoscopic combined intrarenal surgery (ECIRS) combines antegrade percutaneous access with retrograde flexible ureteroscopy, allowing complementary access to different regions of the collecting system [10,11,12,13,14,15]. The retrograde component may facilitate access to migrated fragments or calyces that are difficult to reach through a single percutaneous tract and allows systematic endoscopic inspection of the collecting system before completion of the procedure [10,11,12,13,14,15]. When a vacuum-assisted ureteral access sheath is used, continuous suction additionally provides a route for evacuation of irrigation fluid, stone dust, and small fragments [16,17]. Contemporary experience with suction-assisted flexible ureteroscopy for lower pole stones further supports the rationale for active fragment evacuation [18]. However, suction-source pressure should not be considered equivalent to intrarenal pressure, and any physiological effect of suction on intrarenal pressure requires direct in vivo measurement.

The same laser platform, fiber diameter, and fragmentation settings were used in both treatment periods in our institution. Identical equipment and settings do not, however, imply equivalent fragmentation performance, because delivered energy, fiber route, access, visibility, and the handling of migrated fragments may still have differed. Any contribution of suction-assisted fragment evacuation should therefore be regarded as a separate mechanistic hypothesis that observational data cannot isolate.

Although the feasibility of combining micro-PCNL with flexible ureteroscopy has been reported [12], comparative evidence regarding vacuum-assisted micro-ECIRS and standard micro-PCNL for symptomatic 1–2 cm lower pole stones remains limited. Standard micro-PCNL was chosen as the comparator because it was the institutional standard for these stones before the combined technique was adopted and because both procedures share the same micro-percutaneous access system and laser platform; the comparison therefore reflects the addition of simultaneous retrograde flexible ureteroscopy and suction-assisted evacuation to an otherwise similar antegrade approach. Building on this combined approach, we added retrograde vacuum-assisted flexible ureteroscopy to micro-PCNL with the aim of improving fragment clearance while preserving the minimal invasiveness of the micro-percutaneous tract and reducing the risk of complications related to impaired drainage. The contribution of the present study is to describe stone clearance and complication outcomes after institutional adoption of this combined technique, compared with standard micro-PCNL and assessed on postoperative NCCT at three residual-fragment thresholds in all patients, and thereby to inform whether vacuum-assisted micro-ECIRS merits consideration as a treatment option for 1–2 cm lower pole stones, for which the optimal approach remains unsettled. It does not, however, address how micro-ECIRS compares with contemporary suction-assisted RIRS or mini-PCNL. We therefore evaluated stone clearance and perioperative outcomes following institutional adoption of vacuum-assisted micro-ECIRS, using patients treated with standard micro-PCNL during the earlier institutional treatment period as a historical comparator. Complete stone clearance (Grade A) and cumulative thresholds of no residual fragment > 2 mm (Grade B) and >4 mm (Grade C) were reported; Grade C threshold attainment on postoperative NCCT was the main outcome of the retrospective analysis. Because treatment technique was closely linked to calendar era, this study was designed and interpreted as a retrospective non-concurrent observational comparison of treatment periods rather than as an estimate of an isolated technique-specific treatment effect.

## 2. Materials and Methods

### 2.1. Study Design and Patient Population

This retrospective non-concurrent cohort study was conducted at Kayseri City Hospital and included consecutive adult patients who underwent standard micro-percutaneous nephrolithotomy (micro-PCNL) or vacuum-assisted micro-endoscopic combined intrarenal surgery (micro-ECIRS) for symptomatic 1–2 cm lower pole renal stones between January 2016 and July 2025. Cases were identified from institutional operating-room records and electronic medical records, which covered the entire study period without changes in hospital organization or in the electronic record system. During the study period, a micro-percutaneous approach was the institutional preference for symptomatic 1–2 cm lower pole stones, because it is less invasive than mini-PCNL [9] and because RIRS achieves lower clearance of lower pole stones [2,3,4]. Patients treated with RIRS or mini-PCNL during the same period were not part of the study population. Of 94 patients identified (46 micro-ECIRS and 48 micro-PCNL), 13 were excluded because the 1-month NCCT was unavailable (micro-ECIRS 3, micro-PCNL 7) or clinical data were incomplete (micro-ECIRS 1, micro-PCNL 2). A total of 81 patients were included: 39 underwent standard micro-PCNL and 42 underwent vacuum-assisted micro-ECIRS. The study was reported in accordance with the Strengthening the Reporting of Observational Studies in Epidemiology (STROBE) statement [19]; the completed checklist is provided in Supplementary Table S1.

The two cohorts were largely non-concurrent and represented different institutional treatment periods. Of the 39 standard micro-PCNL procedures, 33 were performed before January 2021. The remaining six were performed after January 2021 (four in 2022 and two in 2023; annual distribution in Supplementary Table S2) because the single-use flexible ureteroscope or the vacuum-assisted ureteral access sheath required for micro-ECIRS was temporarily unavailable owing to supply constraints. All 42 vacuum-assisted micro-ECIRS procedures were performed from January 2021 onward following institutional adoption of the combined technique. Apart from the surgical technique, institutional protocols for preoperative imaging, antimicrobial prophylaxis, postoperative analgesia, discharge criteria, and the recording of operative times did not change during the study period.

Accordingly, treatment technique was closely linked to calendar era and equipment availability. The study was therefore interpreted as an observational comparison of outcomes across two institutional treatment periods rather than as an estimate of an isolated technique-specific treatment effect.

All antegrade percutaneous procedures were performed by the same principal surgeon. Standard micro-PCNL had been performed by this surgeon since 2014, and approximately 40 micro-PCNL procedures had been completed before the present study cohort commenced in 2016. Flexible ureteroscopy had also been routinely performed as an independent procedure for several years before the introduction of vacuum-assisted micro-ECIRS in January 2021. Thus, the combined technique was introduced after established experience with both component procedures. However, no formal learning-curve analysis specific to micro-ECIRS was performed, and an adoption-related learning effect cannot be excluded.

Vacuum-assisted micro-ECIRS was performed using a two-surgeon approach. The principal surgeon performed the antegrade percutaneous component, while a second dedicated urologist simultaneously managed the retrograde flexible ureteroscopic component. The same second surgeon, an endourologist who had performed more than 500 flexible ureteroscopic procedures before adoption of micro-ECIRS, participated throughout the micro-ECIRS period.

### 2.2. Eligibility Criteria and Treatment Pathways

Patients were eligible if they were aged ≥18 years and had a symptomatic, solitary, radiopaque lower pole renal stone measuring 1–2 cm on noncontrast computed tomography (NCCT), with complete clinical and imaging data available. Symptomatic disease was defined as flank pain, macroscopic or microscopic hematuria, or recurrent urinary tract infection attributable to the stone.

**Figure 1:**
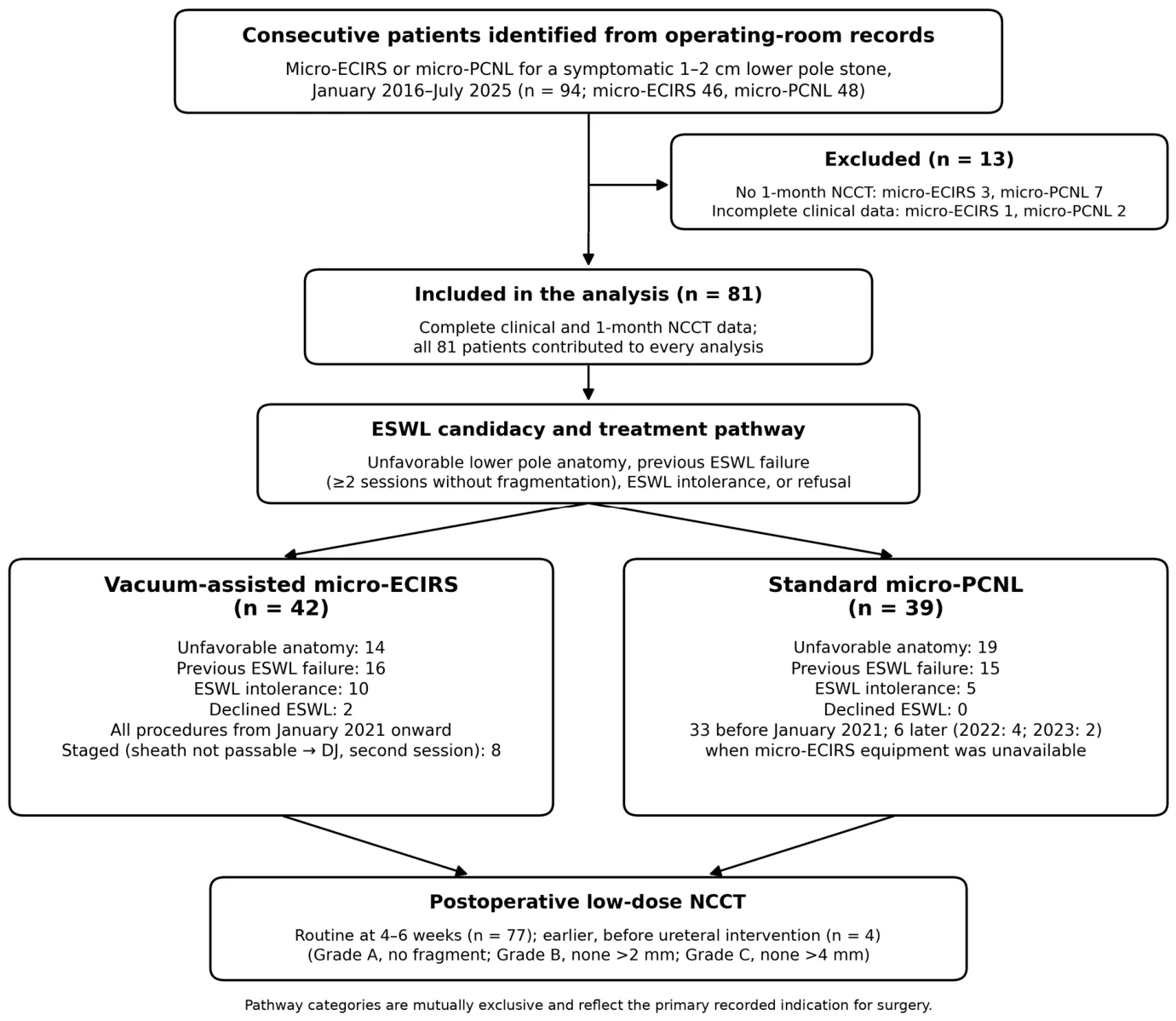
Patient flow and treatment pathways. The figure summarizes study eligibility, ESWL candidacy assessment, pathways leading to surgical treatment, treatment-group allocation, and postoperative imaging follow-up. Treatment allocation was nonrandomized and was influenced by clinical pathway, calendar era, and equipment availability. Pathway categories are mutually exclusive and represent the primary recorded indication for surgery. Of the 39 standard micro-PCNL procedures, 33 were performed before January 2021; the remaining six were performed from January 2021 onward when a single-use flexible ureteroscope or vacuum-assisted ureteral access sheath required for micro-ECIRS was unavailable because of supply constraints. Of 94 consecutive patients identified from operating-room records, 13 were excluded because the 1-month NCCT was unavailable (micro-ECIRS 3, micro-PCNL 7) or clinical data were incomplete (micro-ECIRS 1, micro-PCNL 2). Eight micro-ECIRS patients were treated in two stages because the access sheath could not initially be advanced. Postoperative NCCT was obtained routinely at 4–6 weeks (*n* = 77) or earlier, before intervention for an obstructing ureteral fragment (*n* = 4). Patients who were evaluated but not treated with either procedure could not be reconstructed retrospectively. Abbreviations: DJ, double-J stent; ESWL, extracorporeal shock wave lithotripsy; micro-ECIRS, micro-endoscopic combined intrarenal surgery; micro-PCNL, micro-percutaneous nephrolithotomy; NCCT, noncontrast computed tomography.

Lower pole anatomy was assessed with NCCT and intravenous pyelography (IVP). Anatomical features considered unfavorable for extracorporeal shock wave lithotripsy (ESWL) were an infundibulopelvic angle < 30°, an infundibular width < 5 mm, or an infundibular length > 10 mm. Patients could proceed to surgical treatment because of unfavorable lower pole anatomy, previous ESWL failure (defined as absence of stone fragmentation on follow-up imaging after at least two ESWL sessions), inability to tolerate ESWL because of pain, or patient refusal. For Figure 1, these pathways were assigned mutually exclusively according to the primary documented indication for surgery; when more than one criterion applied, the pathway reflects the indication recorded as driving surgical treatment.

In the vacuum-assisted micro-ECIRS cohort, 14 patients were assigned to the unfavorable-anatomy pathway, 16 to previous ESWL failure, 10 to ESWL intolerance, and 2 to ESWL refusal. In the standard micro-PCNL cohort, the corresponding numbers were 19, 15, 5, and 0. In both cohorts, all patients with ≥1 unfavorable anatomical factor were assigned to the unfavorable-anatomy pathway. Because entry into the surgical pathway was not randomized and differed across clinical circumstances, these pathways represent a potential source of channeling bias.

Exclusion criteria were renal anatomical anomaly, coagulopathy, malignancy at the planned access site, multiple calyceal stones, concomitant ureteral stones, radiolucent stones, incomplete clinical or imaging data, and absence of IVP because renal function or contrast allergy precluded contrast administration. Urinary tract infection was managed as a reason to defer surgery rather than as an exclusion criterion: patients with a positive preoperative urine culture received culture-directed antibiotics and underwent surgery only after a sterile urine culture had been obtained. Consequently, no patient was operated on with an active infection, and no patient was excluded for this reason. Renal insufficiency itself was not an automatic exclusion criterion; patients were excluded only when it prevented IVP according to clinical assessment at the time of treatment.

Radiolucent stones were excluded because intraoperative assessment differed fundamentally between the two techniques. Standard micro-PCNL relied substantially on fluoroscopic localization, whereas micro-ECIRS additionally allowed direct retrograde endoscopic visualization of the collecting system. Inclusion of radiolucent stones could therefore have introduced systematic asymmetry in intraoperative stone assessment between groups. IVP was required in addition to NCCT throughout the study period because the lower pole anatomical measurements (infundibulopelvic angle, infundibular width, and infundibular length) used to assess ESWL candidacy were obtained from IVP. Patients in whom IVP could not be performed were therefore ineligible, which may have selectively excluded some patients with impaired renal function or contrast allergy.

Patient selection and treatment pathways are summarized in Figure 1.

### 2.3. Preoperative Assessment

All patients underwent standardized preoperative evaluation including age, sex, age-adjusted Charlson Comorbidity Index (CCI), American Society of Anesthesiologists (ASA) physical status, previous urological surgery, stone laterality, and degree of hydronephrosis. Baseline laboratory parameters included blood urea nitrogen (BUN), serum creatinine, and hemoglobin. Because the eGFR equation used by the hospital laboratory changed over the study period, and to ensure internally consistent renal-function reporting, estimated glomerular filtration rate (eGFR) used in the present analysis was recalculated from age, sex, and serum creatinine using the 2021 race-free CKD-EPI creatinine equation [20] rather than taken directly from legacy laboratory eGFR fields.

Stone characteristics were assessed using NCCT, complemented by IVP for lower pole anatomical assessment. Recorded stone-related variables included maximum axial stone diameter, stone volume calculated using the ellipsoid formula (length × width × height × π/6), stone density in Hounsfield units (HU), and S.T.O.N.E. nephrolithometry score. Stone density was measured on axial NCCT images as the mean attenuation within an elliptical region of interest placed on the largest cross-section of the stone, excluding its margins. The infundibulopelvic angle, infundibular width, and infundibular length were measured by the treating urologist on IVP obtained before treatment to assess ESWL candidacy or to decide on surgery, that is, before the surgical procedure had been selected and before outcomes were known, using the same method throughout the study period. For the present study, these measurements were re-checked and verified by the investigators before data analysis. Each factor was recorded as present or absent according to the thresholds above, and the number of unfavorable factors was calculated for each patient; continuous anatomical measurements were not retained in the study database. Body mass index was also recorded.

Preoperative urine culture was obtained in all patients. Patients with a positive urine culture received culture-directed antibiotic therapy before surgery, and this was recorded for each patient. A cephalosporin was administered at anesthetic induction for perioperative antimicrobial prophylaxis.

### 2.4. Standard Micro-PCNL Technique

All procedures were performed under general anesthesia. With the patient initially in the dorsal lithotomy position, a 6 Fr open-ended ureteral catheter was inserted cystoscopically under fluoroscopic guidance to opacify the pelvicalyceal system. The patient was subsequently repositioned prone on a radiolucent operating table. Retrograde saline injection through the ureteral catheter was used to facilitate calyceal targeting.

Percutaneous access was obtained under fluoroscopic guidance using a 4.85 Fr (16 G) “all-seeing needle” system (PolyDiagnost, Pfaffenhofen, Germany). After direct optical confirmation of entry into the collecting system, the inner stylet was removed and a three-way Tuohy–Borst connector was attached to the outer sheath. A 272 μm laser fiber was introduced through the central working channel, while continuous irrigation was delivered through the lateral channel using a pressure-regulated pump (IP 200, PolyDiagnost) set at 150–200 mmHg. The ureteral catheter provided passive drainage throughout the procedure.

Stone fragmentation was performed using a 35 W holmium:YAG laser system (Quanta System, Samarate, Italy) with standardized settings of 0.8 J and 20 Hz in standard pulse mode. Fragmented material was allowed to pass spontaneously because the micro-PCNL tract did not permit conventional active fragment extraction; no baskets or other extraction devices were used. Completion of fragmentation was assessed fluoroscopically at the end of the procedure.

A ureteral catheter was left in situ at the end of the procedure and was generally removed on postoperative day 1 in uncomplicated cases. In patients with postoperative fever, removal was delayed according to clinical status. Double-J stenting was performed when clinically indicated, including renal colic following catheter removal or postoperative infectious complications.

### 2.5. Vacuum-Assisted Micro-ECIRS Technique

Vacuum-assisted micro-ECIRS was performed as a simultaneous combination of antegrade micro-percutaneous access and retrograde flexible ureteroscopy using a dedicated two-surgeon approach. The principal surgeon performed the antegrade percutaneous component, while a second urologist simultaneously managed the retrograde flexible ureteroscopic component throughout the procedure.

With the patient initially in the dorsal lithotomy position, an 11/13 Fr vacuum-assisted ureteral access sheath (ClearPetra^®^, Well Lead Medical, Guangzhou, China) was inserted cystoscopically under fluoroscopic guidance. A 9 Fr single-use flexible ureteroscope (HugeMed, Shenzhen, China) was then advanced retrogradely through the sheath. The patient was subsequently repositioned to the prone split-leg position, allowing simultaneous antegrade and retrograde access. Before repositioning, the flexible ureteroscope was withdrawn and the access sheath was secured to the urethral catheter; its position was confirmed fluoroscopically before the ureteroscope was reintroduced.

Routine preoperative ureteral stenting was not performed. When the access sheath could not be advanced, a double-J stent was placed and micro-ECIRS was performed at a second session after passive ureteral dilation; at that session, the stent was removed, the vacuum-assisted sheath was inserted, and the procedure was completed as described. This staged approach was required in 8 of 42 patients (19.0%); the first-stage stent placement lasted a median of 8.5 min (range, 5–15). No complications occurred after the first session, and all perioperative data for these patients were obtained from the definitive micro-ECIRS session. No conversions to another procedure and no ureteral injuries were recorded.

Percutaneous access was obtained using the same 4.85 Fr (16 G) all-seeing needle system used in the standard micro-PCNL cohort. Antegrade access was performed under fluoroscopic guidance, with complementary retrograde endoscopic visualization when needed to identify the target calyx and confirm collecting-system entry.

The sheath tip was positioned in the renal pelvis. Continuous suction was applied through the vacuum-assisted ureteral access sheath using an external vacuum-source setting of approximately −100 to −200 mmHg (negative pressure). Within this range, the suction level was adjusted according to endoscopic visibility and calyceal collapse. These values represent the magnitude of negative pressure at the external suction source and should not be interpreted as intrarenal pressure, which was not directly measured. Pump-assisted irrigation was maintained at 150–200 mmHg, as in the standard micro-PCNL cohort. The vacuum-assisted sheath provided continuous outflow of irrigation fluid, stone dust, and small fragments during lithotripsy and retrograde inspection.

Stone fragmentation was performed with the same 35 W holmium:YAG laser system, 272 μm laser fiber, and standardized settings of 0.8 J and 20 Hz in standard pulse mode as in the standard micro-PCNL cohort, either antegradely through the micro-percutaneous working channel or retrogradely through the flexible ureteroscope, depending on the location of the stone and its fragments. The flexible ureteroscope was used simultaneously for retrograde visualization, access to calyces outside the primary antegrade trajectory, and management of migrated fragments when required. Stone dust and small fragments were evacuated through the suction-assisted ureteral access sheath; no baskets or other extraction devices were used. Thus, the principal technical differences between the groups concerned complementary retrograde access, active fragment evacuation, and endoscopic access to migrated or difficult-to-reach fragments rather than the lithotripsy platform itself.

During the procedure, the flexible ureteroscope was used to inspect calyces not readily accessible through the primary micro-percutaneous tract and to identify migrated fragments. At the end of the procedure, systematic retrograde inspection of the collecting system was performed to identify residual fragments that could be reached and managed before completion of the operation. A 6 Fr ureteral catheter was then left in situ and managed as in the standard micro-PCNL cohort; double-J stenting was not performed routinely.

For both treatment groups, operation time was defined as the interval from the beginning of cystoscopy to completion of the entire procedure, including patient repositioning and all procedural steps. Puncture time was defined as the interval from initiation of percutaneous puncture to confirmed collecting-system access. Lithotripsy time was defined as the recorded interval devoted to laser fragmentation. Operation-time start and stop points were taken from the operating-room records and applied identically in both treatment periods. Fluoroscopy time was obtained from the fluoroscopy unit and included all antegrade and retrograde steps. Irrigation volume represented the total recorded irrigation fluid used during the procedure, and hospital stay was calculated as postoperative inpatient days until discharge. In micro-ECIRS, operation time represents elapsed procedural time during simultaneous two-surgeon work and does not represent surgeon-minutes, total operating-room occupancy, or anesthesia time.

### 2.6. Residual-Fragment Assessment and Outcomes

Postoperative stone status was assessed routinely at 1 month (acceptable window, 4–6 weeks; *n* = 77) using low-dose noncontrast computed tomography (NCCT) reconstructed at a slice thickness of 1.5 mm. In four patients (one micro-ECIRS and three micro-PCNL) who developed renal colic caused by a ureteral fragment, NCCT was obtained earlier, within the first postoperative month and before semirigid ureteroscopy; these pre-intervention images were used for residual-fragment classification. The CT scanner changed during the study period, but the same low-dose acquisition protocol and 1.5-mm reconstruction were used throughout. NCCT examinations were interpreted by staff radiologists as part of routine clinical care, and the maximum residual-fragment diameter was obtained from the formal radiology report. The largest fragment dimension was measured on multiplanar reconstructions. Reports describing “dust” or small unmeasurable fragments were classified as residual fragments ≤ 2 mm, that is, as not meeting Grade A but meeting the Grade B and Grade C thresholds. The location of residual fragments was recorded for all patients with fragments > 4 mm (Section 3.2 and Supplementary Table S3); the number and location of smaller residual fragments were not systematically recorded.

Complete stone clearance was defined strictly as no residual fragment (Grade A) [1]. Because postoperative stone clearance studies use different residual-fragment cutoffs, two additional cumulative thresholds were reported to facilitate comparison with the literature: Grade B, no residual fragment > 2 mm; and Grade C, no residual fragment > 4 mm. Accordingly, Grade B included patients with either no residual fragment or residual fragments ≤ 2 mm, whereas Grade C included patients with no residual fragment or residual fragments ≤ 4 mm. Patients with residual fragments > 4 mm did not meet the Grade C threshold. Grades B and C are therefore reported as residual-fragment thresholds rather than as complete stone clearance.

Achievement of the Grade C threshold (no residual fragment > 4 mm) on postoperative NCCT was the main outcome of the retrospective analysis. It was selected before the outcome data were analyzed because fragments of 4 mm or smaller have commonly been regarded as clinically insignificant in the lower pole literature [1]; however, no written statistical analysis plan or prospective protocol existed, and this designation should not be interpreted as prospective prespecification. Grade A complete stone clearance and Grade B threshold attainment were secondary radiological outcomes.

Deliberate masking of the reporting radiologists to the surgical technique could not be verified retrospectively. To address this, all postoperative NCCT examinations used for classification were subsequently reassessed by a radiologist blinded to treatment group and clinical outcome, who remeasured the largest residual fragment on multiplanar images. The blinded reassessment agreed completely with the original reports for Grade A, Grade B, and Grade C classification (81/81; Cohen’s kappa = 1.00), so the analyses based on the original reports are unchanged. The investigators assigned the radiological categories according to the Grade A status and Grade B/C residual-fragment thresholds using the residual-fragment measurements documented in the formal radiology reports.

Secondary puncture was defined as an additional percutaneous puncture required to access a calyx that could not be adequately reached through the initial micro-percutaneous tract.

Auxiliary procedures were defined as any subsequent stone-related intervention for residual or migrated fragments: semirigid ureteroscopy for an obstructing ureteral fragment, flexible ureterorenoscopy, or mini-PCNL. Semirigid ureteroscopy with double-J stenting for renal colic caused by a ureteral fragment was performed within the first postoperative month, after the pre-intervention NCCT, whereas flexible ureterorenoscopy and mini-PCNL were performed after the 1-month NCCT and within 3 months of the index procedure. In every case, the NCCT used for classification preceded the auxiliary procedure; therefore, Grade A status and Grade B/Grade C threshold attainment reflected the outcome of the index procedure alone. Auxiliary procedures were identified from institutional records only; procedures performed at other centers would not have been captured.

Postoperative pain was assessed on the morning of postoperative day 1 by physicians using a 0–10 visual analogue scale (VAS; 0, no pain; 10, worst imaginable pain). According to the institutional protocol, intravenous paracetamol was routinely administered approximately 2 h after surgery; subsequent routine postoperative analgesia consisted of paracetamol or a nonsteroidal anti-inflammatory drug, and no opioid analgesics were used; this protocol did not change over the study period. Individual analgesic agents and doses were not systematically recorded, so analgesic exposure could not be compared between groups, and postoperative VAS was analyzed as a direct postoperative pain measure.

Renal function parameters included recalculated eGFR, BUN, and serum creatinine; hemoglobin was also assessed preoperatively and on postoperative day 1. Postoperative complications were classified according to the Clavien–Dindo classification [21], with each patient counted once according to the highest complication grade experienced. Complications were recorded for 30 days after surgery using scheduled follow-up visits and hospital records, including outpatient and emergency department attendances. Double-J stents for postoperative renal colic were inserted under spinal anesthesia, without general anesthesia (Grade IIIa). Transient creatinine elevation was defined as an increase of more than 0.3 mg/dL from baseline that resolved with intravenous hydration (Grade I). The Grade IV event was urosepsis requiring intensive care, with dialysis-requiring acute kidney injury and vasopressor-dependent circulatory failure, that is, multiorgan dysfunction (Grade IVb). In the tabulation of complication types, each patient is listed once under the event that determined the highest grade.

Secondary outcomes included Grade A complete stone clearance, Grade B residual-fragment threshold attainment, operation time, fluoroscopy time, puncture time, lithotripsy time, irrigation volume, hospital stay, secondary puncture, auxiliary procedures, postoperative day 1 VAS, perioperative laboratory changes, and postoperative complications.

Only patients with complete postoperative NCCT assessment were included in the radiological outcome analysis; the exclusions resulting from this requirement are reported in Section 2.1 and Figure 1.

### 2.7. Statistical Analysis

All statistical analyses were performed using IBM SPSS Statistics version 30 (IBM Corp., Armonk, NY, USA), except for the penalized regression analysis, which was performed in R version 4.3.3 (R Foundation for Statistical Computing, Vienna, Austria) with the brglm2 package (version 0.9.2). Because this was a retrospective study including all eligible patients treated during the study period, no a priori sample-size calculation was performed.

Continuous variables were summarized as mean ± standard deviation or median (interquartile range [IQR]; range), according to their distribution; normality was assessed using the Kolmogorov–Smirnov test and visual inspection of histograms, and homogeneity of variance was assessed with Levene’s test. Between-group comparisons of continuous variables were performed using the independent-samples Student’s *t* test or Mann–Whitney U test, as appropriate. Categorical variables were presented as numbers and percentages. Fisher’s exact test was used for all two-by-two comparisons of binary outcomes. The overall Clavien–Dindo grade distribution, including patients without complications, was compared using Pearson’s chi-square test; because several cells were sparse, a Monte Carlo exact *p* value (200,000 permutations) was also calculated.

For selected binary outcomes, including Grade A status, Grade B and Grade C threshold attainment, secondary puncture, auxiliary procedures, and overall complications, risk ratios (RRs) with 95% confidence intervals (CIs) were calculated on the log scale, and absolute risk differences with 95% CIs were calculated using the Newcombe hybrid score method. *p* values for the principal binary comparisons were obtained using Fisher’s exact test. Because the confidence intervals and Fisher’s exact *p* values are derived using different inferential procedures, confidence limits very close to the null value may not correspond exactly to a *p* value of 0.05. For continuous perioperative outcomes, mean differences with 95% CIs were calculated without assuming equal variances (Welch correction). All between-group effect estimates were unadjusted unless stated otherwise.

Because treatment allocation was nonrandomized, baseline characteristics were described using standardized mean differences (SMDs) rather than significance testing. SMDs were calculated from patient-level data. For continuous variables and the S.T.O.N.E. score, the SMD was the difference in means divided by the pooled standard deviation, although medians are reported in the Results. For binary variables, SMDs were based on pooled Bernoulli variance, and for variables with more than two categories (including the ordinal CCI, ASA physical status, and number of unfavorable lower pole anatomical factors), a generalized (Mahalanobis distance-based) SMD was calculated from the category proportions; SMDs were calculated with a custom Python script (version 3.11; Supplementary File S1). SMDs were used to characterize the magnitude of baseline imbalance and were not interpreted as hypothesis tests.

Preoperative and postoperative eGFR, BUN, serum creatinine, and hemoglobin were compared within each group using the Wilcoxon signed-rank test. eGFR was recalculated for all patients using the 2021 race-free CKD-EPI creatinine equation before these analyses. Between-group comparisons of perioperative change in laboratory parameters were performed using the Mann–Whitney U test. Postoperative day 1 VAS scores were compared directly between groups using the Mann–Whitney U test; preoperative-to-postoperative VAS change was not analyzed.

To explore temporal confounding descriptively, the standard micro-PCNL cohort was stratified by calendar period (before January 2021, *n* = 33; January 2021 to July 2025, *n* = 6), and Grade A, Grade B, and Grade C outcomes, secondary puncture, auxiliary procedures, and complications were summarized within these subgroups. Because only six micro-PCNL procedures were performed in the later period, this analysis was descriptive and no formal hypothesis testing or adjusted effect estimation was undertaken.

As a sensitivity analysis for the main outcome, Firth’s penalized logistic regression [22] was fitted with treatment group, stone size, number of unfavorable lower pole anatomical factors, and CCI as covariates. These covariates were chosen on clinical grounds, and their number was limited because only 18 patients did not attain the Grade C threshold. Odds ratios (ORs) are reported with profile penalized-likelihood 95% CIs. This analysis addresses measured baseline imbalance only; because treatment and calendar era overlapped almost completely, it was not intended to, and cannot, remove era-related confounding. Because the eight micro-ECIRS patients treated in two stages had an indwelling double-J stent before the definitive procedure, the main outcome was also summarized after excluding these patients.

Achievement of the Grade C threshold (no residual fragment > 4 mm) was the main outcome. Other analyses were considered secondary or exploratory. No adjustment for multiple comparisons was applied. All statistical tests were two-sided, and a *p* value < 0.05 was considered statistically significant.

Patients with incomplete required clinical or imaging data were excluded, and no imputation was performed. Of the 94 patients identified, 13 (13.8%) were excluded for this reason: 4 of 46 (8.7%) treated with micro-ECIRS and 9 of 48 (18.8%) treated with micro-PCNL. All 81 included patients contributed data to every laboratory, perioperative, and radiological analysis.

## 3. Results

### 3.1. Patient Characteristics

Of the 94 consecutive patients identified, 81 met the eligibility criteria and were included in the analysis (Figure 1): 42 underwent vacuum-assisted micro-ECIRS and 39 underwent standard micro-PCNL. All included patients contributed data to every analysis. In the micro-ECIRS cohort, 8 patients (19.0%) were treated in two stages because the access sheath could not initially be advanced.

Baseline demographic, clinical, and stone-related characteristics are summarized in Table 1. Standardized mean differences demonstrated measurable between-group imbalance, most notably for Charlson Comorbidity Index (SMD = 0.657), stone size (SMD = 0.362), number of unfavorable lower pole anatomical factors (SMD = 0.325), and ASA physical status (SMD = 0.321). A double-J stent was present before the definitive procedure only in the eight staged micro-ECIRS patients (SMD = 0.686). Smaller imbalances were observed for laterality, previous urological surgery, hydronephrosis, and S.T.O.N.E. score.

These differences are best regarded as potential confounders rather than simply as baseline imbalance: comorbidity, stone size, and lower pole anatomy may all influence postoperative stone clearance, so the observed differences in Grade C threshold attainment cannot be attributed solely to treatment technique.

Among the individual anatomical factors, an infundibulopelvic angle < 30°, an infundibular width < 5 mm, and an infundibular length > 10 mm were each less frequent in the micro-ECIRS cohort (Table 1). Body mass index and the proportion of patients treated for a positive preoperative urine culture (16.7% vs. 12.8%) were similar between groups.

### 3.2. Residual-Fragment and Perioperative Outcomes

Perioperative and radiological outcomes are summarized in Table 2. Blinded radiological reassessment yielded identical Grade A, Grade B, and Grade C classifications for all 81 patients. On postoperative NCCT, Grade A complete stone clearance was achieved in 18/42 (42.9%) patients after vacuum-assisted micro-ECIRS and 11/39 (28.2%) after standard micro-PCNL (RR 1.52, 95% CI 0.83–2.80; absolute difference 14.7 percentage points, 95% CI −6.1 to 33.6; *p* = 0.246).

The Grade B threshold (no residual fragment > 2 mm) was met in 30/42 (71.4%) patients after micro-ECIRS compared with 18/39 (46.2%) after micro-PCNL (RR 1.55, 95% CI 1.05–2.28; absolute difference 25.3 percentage points, 95% CI 3.9 to 43.8; *p* = 0.025). The Grade C threshold (no residual fragment > 4 mm), the main outcome, was met in 38/42 (90.5%) and 25/39 (64.1%) patients, respectively (RR 1.41, 95% CI 1.09–1.82; absolute difference 26.4 percentage points, 95% CI 8.2 to 43.1; *p* = 0.007).

Considered non-cumulatively, the residual-fragment distribution after micro-ECIRS and micro-PCNL was as follows: no residual fragment, 18 (42.9%) versus 11 (28.2%); fragments > 0–2 mm, 12 (28.6%) versus 7 (17.9%); fragments > 2–4 mm, 8 (19.0%) versus 7 (17.9%); and fragments > 4 mm, 4 (9.5%) versus 14 (35.9%) (Supplementary Table S4). The between-group difference was therefore concentrated in the proportion of patients with fragments > 4 mm. In the Firth sensitivity analysis adjusted for stone size, number of unfavorable lower pole anatomical factors, and CCI, micro-ECIRS remained associated with Grade C threshold attainment (OR 7.30, 95% profile penalized-likelihood CI 1.65–45.34; *p* = 0.008; unadjusted Firth OR 4.86, 95% CI 1.61–17.45) (Supplementary Table S5). Because treatment and calendar era overlapped almost completely, this adjusted estimate does not address era-related confounding. After exclusion of the eight micro-ECIRS patients treated in two stages, Grade C threshold attainment was 31/34 (91.2%) in the remaining micro-ECIRS patients versus 25/39 (64.1%) after micro-PCNL (*p* = 0.011); it was 7/8 (87.5%) in the staged patients.

The need for a secondary percutaneous puncture was lower after micro-ECIRS than after micro-PCNL (2/42 [4.8%] vs. 11/39 [28.2%]; RR 0.17, 95% CI 0.04–0.71; absolute difference −23.4 percentage points, 95% CI −39.4 to −7.4; *p* = 0.006). Similarly, subsequent auxiliary procedures for residual stones were required in 2/42 (4.8%) patients in the micro-ECIRS cohort and 10/39 (25.6%) in the micro-PCNL cohort (RR 0.19, 95% CI 0.04–0.80; absolute difference −20.9 percentage points, 95% CI −36.7 to −5.3; *p* = 0.011). Auxiliary procedures comprised semirigid ureteroscopy with double-J stenting for an obstructing ureteral fragment (1 vs. 3), flexible ureterorenoscopy (1 vs. 4), and mini-PCNL (0 vs. 3), all in patients who had not met the Grade C threshold; their indications, timing, and the locations of all residual fragments > 4 mm are given in Supplementary Table S3. The remaining patients with fragments > 4 mm (2 after micro-ECIRS and 4 after micro-PCNL) were lost to follow-up after the 1-month NCCT, so auxiliary-procedure rates may underestimate the need for further treatment.

Recorded operation time was shorter in the micro-ECIRS cohort (46.7 ± 6.9 vs. 52.8 ± 9.4 min; mean difference −6.15 min, 95% CI −9.84 to −2.47; *p* = 0.001), as was fluoroscopy time (31.6 ± 8.0 vs. 38.4 ± 8.4 s; mean difference −6.74 s, 95% CI −10.39 to −3.09; *p* < 0.001). When the first-stage stent placement was added for the 8 staged patients, micro-ECIRS operation time was 48.3 ± 7.3 min (mean difference −4.51 min, 95% CI −8.27 to −0.75). Puncture time and lithotripsy time did not differ statistically between groups. Irrigation volume and hospital stay were also similar.

### 3.3. Renal Function, Hemoglobin, and Postoperative Pain

Perioperative laboratory parameters and postoperative day 1 pain scores are summarized in Table 3. Using eGFR values recalculated from age, sex, and serum creatinine with the 2021 CKD-EPI equation, no significant within-group changes were observed in eGFR, BUN, or serum creatinine in either cohort. The magnitude of perioperative change in these renal function parameters also did not differ between groups.

Hemoglobin decreased significantly from baseline in both cohorts (both *p* < 0.001). However, the magnitude of perioperative hemoglobin change was not significantly different between vacuum-assisted micro-ECIRS and standard micro-PCNL (*p* = 0.52). The median paired change in hemoglobin was −0.55 g/dL (IQR −1.50 to −0.10) after micro-ECIRS and −1.00 g/dL (IQR −1.60 to 0.00) after micro-PCNL, and the corresponding changes in serum creatinine were 0.02 mg/dL (IQR −0.03 to 0.10) and 0.04 mg/dL (IQR −0.06 to 0.15) (Supplementary Table S6). Creatinine-based eGFR is unreliable during acute, non-steady-state changes in renal function, and these postoperative day 1 data do not establish equivalent or long-term renal safety.

Postoperative pain was evaluated directly using the postoperative day 1 VAS score. Median VAS was 2 (IQR 1–2; range, 1–8) after micro-ECIRS and 3 (IQR 2–3; range, 1–7) after micro-PCNL (*p* < 0.001). Individual analgesic consumption was not available for comparison; preoperative-to-postoperative VAS change was not analyzed.

### 3.4. Postoperative Complications

Postoperative complications are summarized in Table 4. At least one complication occurred within 30 days in 5/42 (11.9%) patients after vacuum-assisted micro-ECIRS and in 12/39 (30.8%) after standard micro-PCNL (RR 0.39, 95% CI 0.150–0.998; absolute difference −18.9 percentage points, 95% CI −35.9 to −1.0; Fisher’s exact *p* = 0.055). The confidence intervals and the Fisher *p* value come from different procedures and lie on either side of the conventional threshold; the comparison is therefore inconclusive, and these data neither establish a difference in overall postoperative morbidity nor demonstrate equivalent safety.

Most complications in both groups were Clavien–Dindo Grade I or II. The overall distribution of Clavien–Dindo grades, including patients without complications, did not differ significantly between groups (Pearson chi-square *p* = 0.31; Monte Carlo exact *p* = 0.37). Classified by the event determining each patient’s highest grade, postoperative fever > 38.5 °C was the principal complication in 3/42 (7.1%) patients after micro-ECIRS and 5/39 (12.8%) after micro-PCNL; antibiotic-requiring urinary tract infection in 1/42 (2.4%) and 2/39 (5.1%); and double-J stent placement for postoperative renal colic in 1/42 (2.4%) and 3/39 (7.7%). In the micro-PCNL cohort, one patient developed transient creatinine elevation (Grade I) and one developed urosepsis with dialysis-requiring acute kidney injury and vasopressor-dependent circulatory failure (Grade IVb); neither event was recorded in the micro-ECIRS cohort.

Because individual complication subtypes were infrequent, no separate effect estimates or hypothesis tests were performed for these events. Complication subtype frequencies are therefore presented descriptively.

### 3.5. Descriptive Calendar-Era Sensitivity Analysis Within Standard Micro-PCNL

To explore whether the standard micro-PCNL results differed materially across calendar periods, that cohort was stratified descriptively into procedures performed before January 2021 (*n* = 33) and those performed from January 2021 to July 2025 (*n* = 6; four in 2022 and two in 2023). Grade C threshold attainment was numerically higher in the later than in the earlier micro-PCNL subgroup (5/6 [83.3%] vs. 20/33 [60.6%]); Grade A and Grade B attainment, secondary puncture, auxiliary procedures, and complications in the two subgroups are shown in Table 5.

Because only six standard micro-PCNL procedures were performed in the later period, this sensitivity analysis was descriptive only. The numerically higher Grade C attainment in the later micro-PCNL subgroup is compatible with a calendar-era effect, but the subgroup is too small for reliable interpretation.

## 4. Discussion

The optimal management of symptomatic 1–2 cm lower pole renal stones remains a clinical challenge because treatment success in this anatomically dependent location depends not only on effective stone fragmentation but also on the subsequent evacuation of fragmented material. Standard micro-PCNL preserves the advantages of direct percutaneous access within a highly miniaturized platform; however, its limited capacity for active fragment extraction, reliance on passive drainage, and restricted ability to access fragments migrating away from the initial percutaneous trajectory may be particularly relevant in the lower pole. The present study evaluated outcomes following institutional adoption of vacuum-assisted micro-ECIRS as an alternative to standard micro-PCNL. Attainment of the Grade B (no fragment > 2 mm) and Grade C (no fragment > 4 mm) thresholds was higher after micro-ECIRS, whereas Grade A complete stone clearance did not differ significantly, and complete clearance was achieved in fewer than half of the micro-ECIRS patients. As a reminder, Grade A denotes no residual fragment, whereas Grades B and C are cumulative residual-fragment thresholds (no fragment > 2 mm and >4 mm, respectively). Secondary punctures and subsequent auxiliary procedures were less frequent, and recorded operation and fluoroscopy times were shorter in the micro-ECIRS cohort. These findings, however, must be interpreted in the context of the non-concurrent study design, the close relationship between treatment technique and calendar era, and measurable baseline imbalance between the two cohorts.

The distinction among these radiological categories is important when interpreting the findings. The differences between groups were concentrated in the proportion of patients with fragments larger than 4 mm, whereas the difference in complete clearance was smaller and not statistically significant. Complete stone clearance is most strictly defined as no residual fragment [1], whereas residual-fragment cutoffs vary across published series. Reporting all three cumulative categories therefore permits comparison with studies using different postoperative clearance thresholds without labeling ≤2-mm or ≤4-mm fragments as completely stone-free.

As noted in the Introduction, identical laser settings do not imply equivalent fragmentation performance, particularly because in micro-ECIRS, the fiber could be used through either route. The higher Grade B and Grade C threshold attainment may therefore reflect several components of the combined procedure, of which suction-assisted evacuation is only one, and the nonsignificant Grade A difference does not indicate equivalent fragmentation efficacy.

The technical configuration of micro-ECIRS provides several potential explanations for the observed residual-fragment pattern. Continuous suction through the vacuum-assisted ureteral access sheath provides a pathway for removal of irrigation fluid, stone dust, and small fragments during lithotripsy. At the same time, retrograde flexible ureteroscopy allows access to fragments that migrate into calyces that cannot be readily reached through the initial micro-percutaneous trajectory and permits systematic inspection of the collecting system before procedure completion. These characteristics are consistent with the broader ECIRS literature, which has emphasized the complementary roles of antegrade and retrograde access in improving visualization, fragment management, and access to anatomically difficult calyces [10,11,12,13,14,15].

More recent evidence also supports these individual components of the combined approach, although the techniques and study populations are not identical to those of the present cohort. A multicenter randomized trial evaluating a suction ureteral access sheath during RIRS provides evidence relevant to active fragment evacuation [23]. Similarly, ureteroscopy-assisted puncture during ECIRS has been associated with improved access-related procedural outcomes [24], while meta-analytic evidence comparing ECIRS with PCNL for complex renal stones, a population distinct from solitary 1–2 cm lower pole stones, supports the broader concept that combined antegrade–retrograde access may improve stone clearance and reduce the need for additional procedures in selected settings [25]. These studies provide mechanistic and clinical context but should not be interpreted as direct evidence for vacuum-assisted micro-ECIRS in 1–2 cm lower pole stones.

The lower frequency of secondary puncture observed after micro-ECIRS is also clinically relevant. Retrograde flexible ureteroscopy may allow migrated stones or fragments to be approached without creating an additional percutaneous tract and may also assist in selecting or confirming the most appropriate calyx for antegrade access. Ureteroscopic assistance during percutaneous access has previously been described as a means of improving access accuracy and reducing additional puncture requirements [24]. Nevertheless, because puncture strategy was not randomized and the two cohorts were treated during different periods, the present data cannot determine how much of the observed difference was attributable specifically to retrograde assistance.

The need for an auxiliary procedure was also lower after micro-ECIRS. Importantly, all auxiliary procedures followed the NCCT used for classification; therefore, they did not contribute to Grade A complete stone clearance or Grade B/Grade C threshold attainment. The lower subsequent intervention rate is compatible with the higher Grade B and Grade C threshold attainment observed at the index postoperative assessment. However, the clinical course of small residual fragments is uncertain: fragments ≤ 4 mm may later cause symptoms, grow, or require intervention, and the present study did not collect longer-term information on these events; moreover, six patients with fragments > 4 mm were lost to follow-up after the 1-month NCCT. Auxiliary procedures were captured only from institutional records and within the early postoperative months, so the two cohorts may not have had identical opportunities for further treatment to be recorded.

Recorded operation time and fluoroscopy time were shorter after micro-ECIRS despite the addition of simultaneous retrograde instrumentation. One possible explanation is that retrograde access allowed migrated fragments to be managed without additional percutaneous punctures and enabled direct endoscopic inspection at procedure completion. However, these findings should not be interpreted as demonstrating greater overall procedural or resource efficiency. Micro-ECIRS required a second dedicated surgeon, a flexible ureteroscope, and a vacuum-assisted access sheath, and total operating-room time, anesthesia time, surgeon-minutes, device expenditure, and downstream costs were not available.

The suction component of the technique warrants similarly cautious interpretation. Previous studies of suction-assisted ureteroscopy have reported improved fragment evacuation and favorable intraoperative visualization [16,17,23]. Continuous suction may also modify collecting-system fluid dynamics. However, the external suction-source setting used in the present study cannot be considered equivalent to intrarenal pressure. Intrarenal pressure and temperature were not directly measured, and no conclusion can therefore be drawn regarding the physiological effect of the vacuum-assisted system on intrarenal pressure, pyelovenous backflow, or thermal conditions. Experimental and clinical studies have established the relevance of intrarenal pressure during upper urinary tract endourological procedures [26], but the present data cannot demonstrate a pressure-mediated mechanism.

Shi et al. previously reported the feasibility of combining micro-PCNL with flexible ureteroscopy in 28 patients with symptomatic, refractory 1–2 cm lower calyceal stones, with stone clearance (no fragment or fragments < 4 mm) of 92.9% at 30 days and 96.4% at 3 months [12]. In that series, micro-PCNL was added under ultrasound guidance in the oblique supine position when retrograde lithotripsy proved difficult; drainage was provided through a conventional access sheath, fragments were partly retrieved with a basket, and a double-J stent was left in every patient. Vacuum-assisted suction was not described, no standard micro-PCNL comparator was included, and postoperative stone assessment was based on plain abdominal radiography of the kidneys, ureters, and bladder (KUB) or CT. In contrast, the present study evaluated a combined procedure planned from the outset, with simultaneous two-surgeon access in the prone split-leg position and continuous suction, against a historical micro-PCNL cohort, using low-dose NCCT and three cumulative residual-fragment thresholds. In a randomized study of lower pole stones ≤ 15 mm, Kandemir et al. reported 3-month complete stone-free rates of 83.3% after microperc and 86.7% after RIRS, with no significant difference between groups [27]. Jiang et al. similarly reported overall stone-free rates of 84.5% after micro-PCNL and 79.3% after flexible ureterorenoscopy for lower calyceal stones ≤ 2 cm, with comparable complication and hospital-stay outcomes [28]. Differences in imaging timing, residual-fragment definitions, patient selection, and operative technique preclude direct numerical comparison; nevertheless, these studies place the present findings within the range reported for contemporary micro-percutaneous and combined endoscopic strategies.

A combined percutaneous–retrograde procedure was used instead of RIRS alone because, in patients with unfavorable lower pole anatomy, a steep infundibulopelvic angle or a narrow or long infundibulum can limit ureteroscope deflection and fragment clearance from the lower calyx, whereas a micro-percutaneous tract provides a direct route to the stone. From a practical standpoint, patients with 1–2 cm lower pole stones and unfavorable lower pole anatomy may theoretically be those most likely to benefit from adding retrograde access and suction to micro-percutaneous lithotripsy. The present retrospective data, however, cannot establish formal selection criteria. Nor do they compare micro-ECIRS with contemporary suction-assisted RIRS or mini-PCNL, which remain reasonable alternatives in this stone-size range. Finally, the findings describe a change in outcomes within a single institution after a new treatment strategy was introduced; they should not be read as evidence supporting broad adoption of the procedure.

Postoperative day 1 VAS scores were lower in the micro-ECIRS cohort. Although the same non-opioid analgesic protocol (paracetamol or a nonsteroidal anti-inflammatory drug) was used throughout the study period, individual analgesic consumption was not recorded, so a contribution of differences in analgesic exposure cannot be excluded. The finding also represents a direct postoperative comparison rather than a change-from-baseline analysis, and a one-point difference in median VAS may be of limited clinical relevance and does not establish a reduced analgesic requirement. Accordingly, postoperative pain should be regarded as a secondary observational finding.

Overall postoperative complications were less frequent after micro-ECIRS, but the comparison was inconclusive (Fisher’s exact *p* = 0.055, with confidence limits close to the null value). These data therefore do not establish a difference in overall postoperative morbidity, and the absence of statistical significance should not be interpreted as equivalent safety. Individual complication types were too infrequent for reliable comparison. Although continuous suction provides a plausible physiological rationale for improved drainage during surgery, the present study cannot establish that it reduces infectious complications or that any observed complication difference was mediated through intrarenal pressure.

The descriptive calendar-era analysis does not provide reassurance against temporal confounding: Grade C attainment was numerically higher in the six later micro-PCNL procedures than in the earlier period (5/6 vs. 20/33), although this subgroup is too small for interpretation. The association with micro-ECIRS persisted in the Firth sensitivity analysis after adjustment for stone size, lower pole anatomy, and comorbidity, but neither analysis can separate the technique from the calendar era in which it was used.

### Limitations

Several limitations merit acknowledgment. First, this was a single-center, nonrandomized, retrospective non-concurrent cohort study in which treatment technique was closely associated with calendar era. Technique-related effects therefore cannot be separated from temporal changes in surgical judgment, institutional practice, or equipment availability, and neither the six later micro-PCNL procedures nor the adjusted Firth model can resolve this. Measurable baseline differences in CCI, stone size, ASA physical status, and unfavorable lower pole anatomy are potential confounders, and other relevant covariates may have been unmeasured.

Second, although the principal surgeon had substantial experience with micro-PCNL before commencement of the study cohort and had independently performed flexible ureteroscopy before adoption of micro-ECIRS, no formal learning-curve analysis of the combined micro-ECIRS technique was undertaken. An adoption-related learning effect therefore cannot be excluded.

Third, the analysis was restricted to patients with complete clinical and 1-month NCCT data. Thirteen of 94 identified patients were excluded for this reason, and exclusion was more frequent in the historical micro-PCNL cohort (18.8% vs. 8.7%). The requirement for IVP may also have excluded some patients with impaired renal function or contrast allergy, and patients treated with RIRS or mini-PCNL during the same period were not studied. This complete-case selection limits what can be concluded about all treated patients and may have influenced the between-group comparison.

Fourth, the sample size was modest, particularly for infrequent secondary outcomes such as individual postoperative complications. No a priori sample-size calculation was performed because all eligible patients treated during the study period were included. Secondary and exploratory analyses were not adjusted for multiple comparisons and should therefore be interpreted cautiously.

Fifth, postoperative stone assessment was based on routine 1-month low-dose NCCT reconstructed at a 1.5-mm slice thickness. Examinations were interpreted by staff radiologists as part of routine clinical care, and residual-fragment measurements from formal reports were used for Grade A, Grade B, and Grade C classification. Deliberate masking of radiologists to treatment technique could not be verified retrospectively; although routine clinical reports are not equivalent to a standardized research assessment, a subsequent blinded reassessment of all examinations by a single radiologist gave identical classifications. Inter-reader variability among multiple blinded readers was not assessed. In addition, auxiliary procedures were captured only from institutional records and complications only for 30 days, so the long-term course of residual fragments, including symptoms, stone growth, and repeat intervention, remains unknown; six patients with fragments > 4 mm (2 after micro-ECIRS and 4 after micro-PCNL) were lost to follow-up after the 1-month NCCT. Imaging time was also not uniform, because four NCCT examinations were obtained earlier than 4–6 weeks when intervention for a ureteral fragment was clinically indicated.

Sixth, the study evaluated a treatment package rather than suction alone. Micro-ECIRS combined suction with simultaneous flexible ureteroscopy, a different position, systematic endoscopic inspection, a second experienced surgeon, and, in 8 patients, staged treatment after passive ureteral dilation. The contribution of each component cannot be isolated, and the observed differences should not be attributed specifically to suction.

Seventh, intrarenal pressure and intrarenal temperature were not directly measured. Accordingly, although continuous suction through the vacuum-assisted ureteral access sheath provides a plausible mechanism for altering intrarenal fluid dynamics, the present study cannot determine whether micro-ECIRS resulted in lower intrarenal pressure, reduced pyelovenous backflow, or altered thermal conditions.

Eighth, individual analgesic agents and doses were not systematically recorded; therefore, the lower postoperative day 1 VAS score observed after micro-ECIRS cannot be interpreted independently of analgesic exposure or translated into a difference in analgesic requirement. Similarly, total operating-room time, anesthesia time, surgeon-minutes, device costs, and downstream treatment costs were unavailable. The study therefore cannot establish overall procedural or economic efficiency, particularly because micro-ECIRS required a dedicated second surgeon, a flexible ureteroscope, and a vacuum-assisted access sheath.

Finally, radiolucent stones were excluded because of the asymmetry that their inclusion would have created in intraoperative stone assessment between fluoroscopy-dependent standard micro-PCNL and micro-ECIRS with supplementary retrograde endoscopic visualization. This restricts generalizability to radiopaque stones. Stone composition data were also unavailable. In addition, fluoroscopy dose metrics such as dose-area product were not systematically recorded; fluoroscopy time should therefore be regarded only as an indirect measure of radiation exposure. The resource requirements of the two-surgeon micro-ECIRS approach may further limit generalizability to centers without comparable endourological infrastructure.

## 5. Conclusions

In this retrospective, predominantly non-concurrent cohort, institutional adoption of vacuum-assisted micro-ECIRS for symptomatic 1–2 cm lower pole renal stones was associated with higher rates of no residual fragment larger than 2 mm (Grade B) or 4 mm (Grade C) and fewer additional punctures and subsequent procedures. A difference in complete stone clearance (Grade A, no residual fragment) was not demonstrated. Grades B and C are cumulative residual-fragment thresholds rather than measures of complete clearance.

Treatment–era confounding, baseline imbalance, and complete-case selection prevent causal attribution to the technique. Adequately powered prospective concurrent or randomized studies incorporating centralized blinded radiological assessment, direct intrarenal pressure measurement, and formal resource and cost evaluation are warranted.

## Figures and Tables

**Table 1:** Baseline demographic, clinical, and stone-related characteristics.

Variable	Vacuum-Assisted Micro-ECIRS (*n* = 42)	Standard Micro-PCNL (*n* = 39)	SMD
Age, years, median (IQR; range)	49 (43–56; 32–70)	52 (38.5–61; 25–77)	0.018
Male sex, *n* (%)	28 (66.7)	25 (64.1)	0.054
Body mass index, kg/m^2^, median (IQR; range)	27.5 (22–30.8; 20–33)	25 (22–30; 20–33)	0.142
Charlson Comorbidity Index (age-adjusted)			0.657
0	18 (42.9)	10 (25.6)	
1	14 (33.3)	12 (30.8)	
2	2 (4.8)	10 (25.6)	
3	6 (14.3)	6 (15.4)	
4	2 (4.8)	1 (2.6)	
ASA physical status			0.321
I	8 (19.0)	7 (17.9)	
II	32 (76.2)	32 (82.1)	
III	2 (4.8)	0	
Laterality			0.162
Left	15 (35.7)	17 (43.6)	
Right	27 (64.3)	22 (56.4)	
Previous urological surgery			0.122
None	30 (71.4)	27 (69.2)	
URS	10 (23.8)	9 (23.1)	
PCNL	2 (4.8)	3 (7.7)	
Hydronephrosis			0.100
None–mild	36 (85.7)	32 (82.1)	
Moderate	6 (14.3)	7 (17.9)	
Unfavorable lower pole anatomical factors, *n*			0.325
0	28 (66.7)	20 (51.3)	
1	7 (16.7)	9 (23.1)	
2	6 (14.3)	8 (20.5)	
3	1 (2.4)	2 (5.1)	
Stone size, mm, median (IQR; range)	16.5 (14–19; 10–20)	18 (15.5–19.5; 10–20)	0.362
Stone volume, mm^3^, median (IQR; range)	1658 (1154–2101; 535–3579)	1560 (1203–2341; 669–3896)	0.059
Stone density, HU, median (IQR; range)	1020 (828–1159; 500–1370)	980 (948–1075; 700–1290)	0.052
S.T.O.N.E. score, median (IQR; range)	6 (5.25–6; 5–8)	6 (6–6.5; 5–8)	0.125
Treated positive preoperative urine culture, *n* (%)	7 (16.7)	5 (12.8)	0.109
Preoperative double-J stent before the definitive procedure, *n* (%)	8 (19.0)	0	0.686
Infundibulopelvic angle < 30°, *n* (%)	12 (28.6)	15 (38.5)	0.211
Infundibular width < 5 mm, *n* (%)	6 (14.3)	10 (25.6)	0.287
Infundibular length > 10 mm, *n* (%)	4 (9.5)	6 (15.4)	0.178

Note: Continuous variables are presented as median (interquartile range [IQR]; range), and categorical variables as *n* (%). Baseline characteristics were described using standardized mean differences rather than hypothesis testing. Generalized SMDs were used for multicategory variables. SMDs were calculated from patient-level means and standard deviations (continuous variables and S.T.O.N.E. score), pooled Bernoulli variance (binary variables), or the generalized Mahalanobis-distance method (variables with more than two categories, including CCI, ASA physical status, and number of unfavorable anatomical factors) and are descriptive measures of baseline imbalance. CCI was age-adjusted. Individual lower pole anatomical factors were recorded as present or absent. No patient received routine preoperative ureteral stenting; the double-J stents shown were placed in eight micro-ECIRS patients at a first-stage session because the access sheath could not be advanced, and the definitive procedure was performed at a second session (Section 2.5). Abbreviations: ASA, American Society of Anesthesiologists; HU, Hounsfield unit; IQR, interquartile range; micro-ECIRS, micro-endoscopic combined intrarenal surgery; micro-PCNL, micro-percutaneous nephrolithotomy; PCNL, percutaneous nephrolithotomy; SMD, standardized mean difference; URS, ureteroscopy.

**Table 2:** Perioperative and postoperative outcomes.

Variable	Vacuum-Assisted Micro-ECIRS (*n* = 42)	Standard Micro-PCNL (*n* = 39)	Effect Estimate (95% CI)	*p* Value
Operation time, min	46.7 ± 6.9	52.8 ± 9.4	MD −6.15 (−9.84 to −2.47)	0.001
Fluoroscopy time, s	31.6 ± 8.0	38.4 ± 8.4	MD −6.74 (−10.39 to −3.09)	<0.001
Puncture time, s	115.8 ± 14.0	121.9 ± 18.0	MD −6.09 (−13.27 to 1.09)	0.095
Lithotripsy time, min	20.1 ± 4.2	22.3 ± 6.2	MD −2.16 (−4.53 to 0.21)	0.073
Irrigation volume, mL, median (IQR; range)	790 (593–1010; 420–1600)	800 (730–870; 550–1500)	—	0.65
Hospital stay, days, median (IQR; range)	2 (1–2; 1–6)	2 (2–3; 1–10)	—	0.09
Secondary puncture, *n* (%)	2 (4.8)	11 (28.2)	RR 0.17 (0.04–0.71); RD −23.4 (−39.4 to −7.4)	0.006
Grade A: complete stone clearance, *n* (%)	18 (42.9)	11 (28.2)	RR 1.52 (0.83–2.80); RD 14.7 (−6.1 to 33.6)	0.246
Grade B: no residual fragment > 2 mm, *n* (%)	30 (71.4)	18 (46.2)	RR 1.55 (1.05–2.28); RD 25.3 (3.9 to 43.8)	0.025
Grade C: no residual fragment > 4 mm, *n* (%)	38 (90.5)	25 (64.1)	RR 1.41 (1.09–1.82); RD 26.4 (8.2 to 43.1)	0.007
Auxiliary procedure, *n* (%)	2 (4.8)	10 (25.6)	RR 0.19 (0.04–0.80); RD −20.9 (−36.7 to −5.3)	0.011

Note: Normally distributed continuous variables are presented as mean ± standard deviation; non-normally distributed variables as median (IQR; range); categorical variables as *n* (%). Grade A denotes complete stone clearance (no residual fragment). Grade B and Grade C are cumulative thresholds of no residual fragment > 2 mm and >4 mm, respectively. RD denotes the absolute risk difference in percentage points (micro-ECIRS minus micro-PCNL; Newcombe hybrid score CI). All effect estimates are unadjusted; mean differences were calculated without assuming equal variances. Grade C threshold attainment was the main outcome of the retrospective analysis. All auxiliary procedures followed the NCCT used for classification (an earlier pre-intervention NCCT in four patients with an obstructing ureteral fragment). Abbreviations: CI, confidence interval; MD, mean difference; micro-ECIRS, micro-endoscopic combined intrarenal surgery; micro-PCNL, micro-percutaneous nephrolithotomy; RD, risk difference; RR, risk ratio; IQR, interquartile range.

**Table 3:** Perioperative laboratory parameters and postoperative day 1 pain.

Variable	Micro-ECIRS Preop	Micro-ECIRS POD1	Within-Group P	Micro-PCNL Preop	Micro-PCNL POD1	Within-Group P	Between-Group P
eGFR, mL/min/1.73 m^2^	97.7 (55.2–120.2)	93.5 (53.6–120.6)	0.055	102.7 (59.0–137.2)	92.2 (37.3–127.4)	0.139	0.992 †
BUN, mg/dL	14 (8–24)	14 (6–24)	0.80	14 (8–30)	14 (8–30)	0.59	0.87 †
Creatinine, mg/dL	0.85 (0.55–1.29)	0.89 (0.55–1.57)	0.083	0.89 (0.52–1.16)	0.90 (0.47–1.53)	0.134	0.824 †
Hemoglobin, g/dL	15.4 (11.0–19.1)	14.5 (11.0–17.5)	<0.001	15.3 (11.2–17.9)	14.4 (8.8–17.6)	<0.001	0.52 †
POD1 VAS score	—	2 (1–8)	—	—	3 (1–7)	—	<0.001 ‡

Note: Values are presented as median (range). Distributions of individual paired changes are provided in Supplementary Table S6. eGFR was recalculated from age, sex, and serum creatinine using the 2021 race-free CKD-EPI creatinine equation. Within-group preoperative-to-postoperative comparisons were performed using the Wilcoxon signed-rank test. † Between-group comparison of perioperative change was performed using the Mann–Whitney U test. ‡ Postoperative day 1 VAS was compared directly between groups using the Mann–Whitney U test. Abbreviations: BUN, blood urea nitrogen; eGFR, estimated glomerular filtration rate; micro-ECIRS, micro-endoscopic combined intrarenal surgery; micro-PCNL, micro-percutaneous nephrolithotomy; POD1, postoperative day 1; VAS, visual analogue scale.

**Table 4:** Postoperative complications and Clavien–Dindo classification.

Variable	Vacuum-Assisted Micro-ECIRS (*n* = 42)	Standard Micro-PCNL (*n* = 39)	Effect Estimate (95% CI)	*p* Value
Overall complications, *n* (%)	5 (11.9)	12 (30.8)	RR 0.39 (0.150–0.998); RD −18.9 (−35.9 to −1.0)	0.055
Clavien–Dindo classification				0.31 *
Grade I	3 (7.1)	6 (15.4)	—	—
Grade II	1 (2.4)	2 (5.1)	—	—
Grade IIIa	1 (2.4)	3 (7.7)	—	—
Grade IIIb	0	0	—	—
Grade IVb	0	1 (2.6)	—	—
Grade V	0	0	—	—
Principal complication type (event determining the highest grade)				
Fever > 38.5 °C	3 (7.1)	5 (12.8)	—	—
Antibiotic-requiring urinary tract infection	1 (2.4)	2 (5.1)	—	—
DJ stent-requiring renal colic	1 (2.4)	3 (7.7)	—	—
Transient creatinine elevation	0	1 (2.6)	—	—
Urosepsis	0	1 (2.6)	—	—

Note: Overall complication rate represents patients experiencing at least one postoperative complication within 30 days. Each patient appears once in the complication-type rows, under the event that determined the highest grade; additional lower-grade events in the same patient are not listed separately. * Pearson chi-square test across all grades including patients without complications (Monte Carlo exact *p* = 0.37). RD, absolute risk difference in percentage points (Newcombe hybrid score CI). Effect estimation was restricted to the overall complication outcome because individual complication subtypes contained few events. Abbreviations: CI, confidence interval; DJ, double-J; micro-ECIRS, micro-endoscopic combined intrarenal surgery; micro-PCNL, micro-percutaneous nephrolithotomy; RD, risk difference; RR, risk ratio.

**Table 5:** Descriptive outcomes within the standard micro-PCNL cohort by calendar period.

Outcome	Before January 2021 (*n* = 33)	January 2021–July 2025 (*n* = 6)	Statistical Comparison
Grade A: complete stone clearance	9/33 (27.3%)	2/6 (33.3%)	Descriptive only
Grade B: no residual fragment > 2 mm	15/33 (45.5%)	3/6 (50.0%)	Descriptive only
Grade C: no residual fragment > 4 mm	20/33 (60.6%)	5/6 (83.3%)	Descriptive only
Secondary puncture	10/33 (30.3%)	1/6 (16.7%)	Descriptive only
Auxiliary procedure	9/33 (27.3%)	1/6 (16.7%)	Descriptive only
Any complication within 30 days	10/33 (30.3%)	2/6 (33.3%)	Descriptive only

Note: Grade A denotes complete stone clearance. Grade B and Grade C denote cumulative thresholds of no residual fragment > 2 mm and >4 mm, respectively. No formal hypothesis testing was undertaken because only six standard micro-PCNL procedures were performed during the later period.

## Data Availability

The data presented in this study are available on request from the corresponding author, including for the purposes of peer review, due to patient privacy and institutional ethical restrictions. The analysis code is provided in the Supplementary Materials (File S1), and additional aggregate results are presented in Supplementary Tables S1–S6.

## Supplementary Materials

The following supporting information can be downloaded at: https://www.mdpi.com/article/10.3390/jcm15218534/s1, Table S1: STROBE checklist; Table S2: Annual distribution of procedures by treatment group; Table S3: Auxiliary procedures and location of residual fragments > 4 mm; Table S4: Non-cumulative distribution of residual fragments on postoperative NCCT; Table S5: Firth penalized logistic regression for Grade C threshold attainment; Table S6: Distribution of individual perioperative changes; File S1: Analysis code (Python and R scripts).

## Abbreviations

ASA, American Society of Anesthesiologists; BUN, blood urea nitrogen; CCI, Charlson Comorbidity Index; CI, confidence interval; CKD-EPI, Chronic Kidney Disease Epidemiology Collaboration; DJ, double-J; ECIRS, endoscopic combined intrarenal surgery; eGFR, estimated glomerular filtration rate; ESWL, extracorporeal shock wave lithotripsy; Fr, French; HU, Hounsfield unit; IQR, interquartile range; IVP, intravenous pyelography; KUB, plain abdominal radiography of the kidneys, ureters, and bladder; micro-ECIRS, micro-endoscopic combined intrarenal surgery; micro-PCNL, micro-percutaneous nephrolithotomy; NCCT, noncontrast computed tomography; OR, odds ratio; PCNL, percutaneous nephrolithotomy; RD, risk difference; RIRS, retrograde intrarenal surgery; RR, risk ratio; SD, standard deviation; SMD, standardized mean difference; S.T.O.N.E., stone size, tract length, obstruction, number of involved calyces, and essence (stone density); URS, ureteroscopy; VAS, visual analogue scale.

## References

[1] European Association of Urology (2026). EAU Guidelines on Urolithiasis. Edn. Presented at the EAU Annual Congress London, United Kingdom 2026.

[2] Donaldson J.F., Lardas M., Scrimgeour D., Stewart F., MacLennan S., Lam T.B.L., McClinton S. (2015). Systematic review and meta-analysis of the clinical effectiveness of shock wave lithotripsy, retrograde intrarenal surgery, and percutaneous nephrolithotomy for lower-pole renal stones. Eur. Urol..

[3] Awedew A.F., Seman Y.S., Yalew D.Z., Wondmeneh Y.C., Yigzaw W.A. (2023). Efficacy and safety of surgical treatment for 1–2 cm sized lower pole of renal stone: Network meta-analysis of randomized control trials. Urolithiasis.

[4] Nedbal C., Somani B.K. (2025). Lower pole stones 1–2 cm: Navigating treatment choices. BJU Int..

[5] Desai M.R., Sharma R., Mishra S., Sabnis R.B., Stief C., Bader M. (2011). Single-step percutaneous nephrolithotomy (Microperc): The initial clinical report. J. Urol..

[6] Ganpule A.P., Chabra J., Desai M.R. (2018). “Microperc” micropercutaneous nephrolithotomy: A review of the literature. Urolithiasis.

[7] Biligere S., Heng C.T., Cracco C., Mangat R., Ong C.S.H., Thandapani K., Inoue T., Sarica K., Sabnis R.B., Desai M. (2021). Tips and tricks to improve ergonomics, efficacy, versatility, and overcome limitations of micro percutaneous nephrolithotomy. Front. Surg..

[8] Tepeler A., Akman T., Silay M.S., Akcay M., Ersoz C., Kalkan S., Armagan A., Sarica K. (2014). Comparison of intrarenal pelvic pressure during micro-percutaneous nephrolithotomy and conventional percutaneous nephrolithotomy. Urolithiasis.

[9] Gao X., Wang W., Peng L., Di X., Xiao K., Chen J., Jin T. (2021). Comparison of micro-percutaneous and mini-percutaneous nephrolithotomy in the treatment of renal stones: A systematic review and meta-analysis. Front. Surg..

[10] Cracco C.M., Scoffone C.M. (2020). Endoscopic combined intrarenal surgery (ECIRS)—Tips and tricks to improve outcomes: A systematic review. Urol. Res. Pract..

[11] Estrade V., Meria P., Almeras C., Lithiasis Committee of the French Association of Urology (CLAFU) (2023). 2022 Recommendations of the AFU Lithiasis Committee: Combined approach for the management of kidney and ureteral stones (Endoscopic Combined IntraRenal Surgery, ECIRS). Prog. Urol..

[12] Shi X.Q., Ma T., Wang W., Cui Z., Li X., Gao Y. (2023). Application of Micro-Percutaneous Nephrolithotomy (Micro-PCNL) combined with FURL in 1–2 cm symptomatic, refractory lower calyceal stones. Pak. J. Med. Sci..

[13] Kang D.H., Jeong J.Y., Moon Y.J., Jung H.D., Kim L., Lee J.Y. (2026). Endoscopic combined intrarenal surgery: From percutaneous nephrolithotomy to the future of stone management. Investig. Clin. Urol..

[14] Nedbal C., Jahrreiss V., Cerrato C., Castellani D., Kamal W.K., Hameed Z., Gauhar V., Juliebø-Jones P., Tzelves L., Kallidonis P. (2023). Current role of endoscopic combined intrarenal surgery in the management of renal stones: A scoping review. Indian J. Urol..

[15] Pellanda A.B., Torricelli F.C.M., Denstedt J., Danilovic A., Marchini G.S., Vicentini F.C., Batagello C.A., Nahas W.C., Mazzucchi E. (2024). Endoscopic Combined Intrarenal Surgery: Best practices and future perspectives. Int. Braz. J. Urol..

[16] Gauhar V., Traxer O., Castellani D., Ragoori D., Heng C.T., Chew B.H., Somani B.K., Bin Hamri S. (2023). A feasibility study on clinical utility, efficacy and limitations of 2 types of flexible and navigable suction ureteral access sheaths in retrograde intrarenal surgery for renal stones. Urology.

[17] Jahrreiss V., Nedbal C., Castellani D., Gauhar V., Seitz C., Zeng G., Juliebø-Jones P., Keller E., Tzelves L., Geraghty R. (2024). Is suction the future of endourology? Overview from EAU Section of Urolithiasis. Ther. Adv. Urol..

[18] Shrestha A., Traxer O., Seitz C., Corrales M., Castellani D., Chew B.H., Ragoori D., Mishra U., Bin Hamri S., Tan K.M. (2025). Assessing flexible ureteroscopy outcomes for lower pole versus non-lower pole stones using the flexible and navigable suction ureteric access sheath: A prospective multicenter study by EAU Endourology and PEARLS group. World J. Urol..

[19] von Elm E., Altman D.G., Egger M., Pocock S.J., Gøtzsche P.C., Vandenbroucke J.P., STROBE Initiative (2007). The Strengthening the Reporting of Observational Studies in Epidemiology (STROBE) statement: Guidelines for reporting observational studies. Lancet.

[20] Inker L.A., Eneanya N.D., Coresh J., Tighiouart H., Wang D., Sang Y., Crews D.C., Doria A., Estrella M.M., Froissart M. (2021). New creatinine- and cystatin C-based equations to estimate GFR without race. N. Engl. J. Med..

[21] Dindo D., Demartines N., Clavien P.A. (2004). Classification of surgical complications: A new proposal with evaluation in a cohort of 6336 patients and results of a survey. Ann. Surg..

[22] Firth D. (1993). Bias reduction of maximum likelihood estimates. Biometrika.

[23] Zhu W., Liu S., Cao J., Wang H., Liang H., Jiang K., Cui Y., Chai C.A., Sahinler E.B., Aquino A. (2024). Tip bendable suction ureteral access sheath versus traditional sheath in retrograde intrarenal stone surgery: An international multicentre, randomized, parallel group, superiority study. eClinicalMedicine.

[24] Taguchi K., Yamashita S., Hamamoto S., Deguchi R., Kawase K., Okada T., Kato T., Ando R., Okada A., Kohjimoto Y. (2021). Ureteroscopy-assisted puncture for ultrasonography-guided renal access significantly improves overall treatment outcomes in endoscopic combined intrarenal surgery. Int. J. Urol..

[25] Liu Y.H., Jhou H.J., Chou M.H., Wu S.T., Cha T.L., Yu D.S., Sun G.H., Chen P.H., Meng E. (2022). Endoscopic Combined Intrarenal Surgery Versus Percutaneous Nephrolithotomy for Complex Renal Stones: A systematic review and meta-analysis. J. Pers. Med..

[26] Tokas T., Skolarikos A., Herrmann T.R.W., Nagele U., Training and Research in Urological Surgery and Technology (T.R.U.S.T.)-Group (2019). Pressure matters 2: Intrarenal pressure ranges during upper-tract endourological procedures. World J. Urol..

[27] Kandemir A., Guven S., Balasar M., Sonmez M.G., Taskapu H., Gurbuz R. (2017). A prospective randomized comparison of micropercutaneous nephrolithotomy (Microperc) and retrograde intrarenal surgery (RIRS) for the management of lower pole kidney stones. World J. Urol..

[28] Jiang K., Chen H., Yu X., Chen Z., Ye Z., Yuan H. (2019). The “all-seeing needle” micro-PCNL versus flexible ureterorenoscopy for lower calyceal stones of ≤2 cm. Urolithiasis.

