# Supplementary material for: Outcomes Following Institutional Adoption of Vacuum-Assisted Micro-ECIRS for Symptomatic 1–2 cm Lower Pole Renal Stones: A Retrospective Non-Concurrent Cohort Study
Source: J Clin Med. 2026 Nov 2;15(21):8534. doi: 10.3390/jcm15218534 (PMC13764316; doi:10.3390/jcm15218534)
Supplement: Supplementary file 1 [file jcm-15-08534-s001.zip › JCM_Supplementary_Material_R1.pdf]

Supplementary Material

Outcomes Following Institutional Adoption of Vacuum-Assisted Micro-ECIRS for Symptomatic 1–2 cm Lower Pole Renal Stones: A Retrospective Non-Concurrent Cohort Study

Supplementary Table S1. STROBE checklist (cohort studies).

Item	No.	Recommendation	Location in manuscript
Title and abstract	1	Indicate design; informative and balanced abstract	Title; Abstract
Background/rationale	2	Scientific background and rationale	Section 1
Objectives	3	Specific objectives	Section 1 (final paragraph)
Study design	4	Key elements of design	Section 2.1
Setting	5	Setting, locations, dates of recruitment, follow-up, data collection	Sections 2.1, 2.6; Institutional Review Board Statement
Participants	6	Eligibility, sources, selection, follow-up methods	Sections 2.1, 2.2; Figure 1
Variables	7	Outcomes, exposures, confounders, effect modifiers	Sections 2.3, 2.6
Data sources/measurement	8	Sources and methods of assessment	Sections 2.3–2.6
Bias	9	Efforts to address potential sources of bias	Sections 2.6, 2.7; Section 4.1
Study size	10	How study size was arrived at	Sections 2.1, 2.7
Quantitative variables	11	Handling of quantitative variables	Section 2.7
Statistical methods	12a–e	Methods, subgroups, missing data, loss to follow-up, sensitivity analyses	Section 2.7
Participants	13	Numbers at each stage; reasons for non-participation; flow diagram	Sections 2.1, 3.1; Figure 1
Descriptive data	14	Characteristics; missing data; follow-up time	Section 3.1; Table 1
Outcome data	15	Numbers of outcome events	Sections 3.2–3.4; Tables 2–4; Supplementary Tables S3 and S4
Main results	16	Unadjusted and adjusted estimates with precision	Section 3.2; Table 2; Supplementary Table S5
Other analyses	17	Subgroup, interaction, sensitivity analyses	Section 3.5; Table 5; Supplementary Tables S2, S5, S6
Key results	18	Summary of key results	Section 4 (first paragraph)
Limitations	19	Limitations, bias, imprecision	Section 4.1
Interpretation	20	Cautious overall interpretation	Sections 4, 5
Generalisability	21	External validity	Section 4.1 (final paragraph)
Funding	22	Funding source and role of funders	Funding statement

Supplementary Table S2. Annual distribution of procedures by treatment group.

Year	Vacuum-assisted micro-ECIRS (n = 42)	Standard micro-PCNL (n = 39)
2016	0	4
2017	0	6
2018	0	7
2019	0	9
2020	0	7
2021	6	0
2022	10	4
2023	9	2
2024	8	0
2025 (January–July)	9	0
Total	42	39

Values are numbers of procedures. Micro-PCNL procedures after 2020 (n = 6) were performed when the single-use flexible ureteroscope or vacuum-assisted ureteral access sheath required for micro-ECIRS was unavailable.

Supplementary Table S3. Auxiliary procedures and location of residual fragments >4 mm.

	Vacuum-assisted micro-ECIRS (n = 42)	Standard micro-PCNL (n = 39)
Patients with residual fragment >4 mm (Grade C not met)	4	14
Auxiliary procedures, total	2	10
Semirigid ureteroscopy and double-J stenting for renal colic caused by a ureteral fragment; within the first postoperative month, after a pre-intervention NCCT	1	3
Flexible ureterorenoscopy; after the 1-month NCCT, within 3 months	1	4
Mini-PCNL; after the 1-month NCCT, within 3 months	0	3
No auxiliary procedure; lost to follow-up after the 1-month NCCT	2	4
Location of the residual fragment >4 mm		
Ureter	1	3
Ureteropelvic junction or renal pelvis	1	4
Lower calyx	2	7

Values are numbers of patients. All auxiliary procedures were performed at the study center in patients who had not met the Grade C threshold, and in every case the NCCT used for classification preceded the procedure. Fragment locations are reported for patients with residual fragments >4 mm only; the number and location of smaller fragments were not systematically recorded. Locations of fragments treated by auxiliary procedures: micro-ECIRS, ureter (n = 1) and ureteropelvic junction (n = 1); micro-PCNL, ureter (n = 3), renal pelvis (n = 3), and lower calyx (n = 4). Locations in patients lost to follow-up: micro-ECIRS, lower calyx (n = 2); micro-PCNL, lower calyx (n = 3) and renal pelvis (n = 1). NCCT, noncontrast computed tomography; PCNL, percutaneous nephrolithotomy.

Supplementary Table S4. Non-cumulative distribution of residual fragments on postoperative NCCT.

Largest residual fragment	Vacuum-assisted micro-ECIRS (n = 42)	Standard micro-PCNL (n = 39)
None (Grade A)	18 (42.9)	11 (28.2)
>0–2 mm	12 (28.6)	7 (17.9)
>2–4 mm	8 (19.0)	7 (17.9)
>4 mm (Grade C not met)	4 (9.5)	14 (35.9)

Values are n (%). Cumulative thresholds: Grade B = first two rows; Grade C = first three rows. Reports describing "dust" or unmeasurable small fragments were classified in the >0–2 mm category.

Supplementary Table S5. Firth penalized logistic regression for Grade C threshold attainment (no residual fragment >4 mm).

Variable	Odds ratio	95% profile penalized-likelihood CI	P value
Unadjusted model: micro-ECIRS vs. micro-PCNL	4.86	1.61–17.45	0.005
Adjusted model: micro-ECIRS vs. micro-PCNL	7.30	1.65–45.34	0.008
Stone size (per 1 mm)	0.85	0.63–1.12	0.25
Unfavorable lower pole anatomical factors (per factor)	0.14	0.04–0.32	<0.001
Age-adjusted CCI (per point)	1.56	0.81–3.21	0.18

n = 81; 18 patients did not attain Grade C. Covariates were selected on clinical grounds and limited in number because of the small number of events. P values from penalized likelihood-ratio tests. Analysis performed in R 4.3.3; Firth estimates were obtained with the brglm2 package (version 0.9.2; type = "AS_mean") and profile penalized-likelihood confidence intervals with a custom script (Supplementary File S1). Treatment and calendar era overlapped almost completely; this model adjusts for measured baseline imbalance only and does not remove era-related confounding. CCI, Charlson Comorbidity Index; CI, confidence interval.

Supplementary Table S6. Distribution of individual perioperative changes (postoperative day 1 minus preoperative).

**Parameter**	**Vacuum-assisted micro-ECIRS (n = 42)**	**Standard micro-PCNL (n = 39)**	**P value***
Hemoglobin, g/dL	−0.55 (−1.50 to −0.10); −4.10 to 2.40	−1.00 (−1.60 to 0.00); −3.10 to 1.70	0.52
Serum creatinine, mg/dL	0.02 (−0.03 to 0.10); −0.26 to 0.30	0.04 (−0.06 to 0.15); −0.50 to 0.50	0.82
eGFR, mL/min/1.73 m²	−1.30 (−10.29 to 1.10); −29.71 to 20.44	−1.98 (−11.62 to 4.32); −42.90 to 30.28	0.99
BUN, mg/dL	0.0 (−1.0 to 0.0); −5.8 to 5.0	0.0 (0.0 to 0.0); −6.0 to 4.0	0.87

Values are median (interquartile range); range of individual paired changes. *Mann-Whitney U test comparing paired changes between groups. eGFR was recalculated with the 2021 race-free CKD-EPI creatinine equation. BUN, blood urea nitrogen; eGFR, estimated glomerular filtration rate.
